# A Self Regulating and Crowdsourced Indoor Positioning System through Wi-Fi Fingerprinting for Multi Storey Building

**DOI:** 10.3390/s18113766

**Published:** 2018-11-04

**Authors:** Soumya Prakash Rana, Javier Prieto, Maitreyee Dey, Sandra Dudley, Juan Manuel Corchado

**Affiliations:** 1 Division of Electrical and Electronic Engineering, School of Engineering, London South Bank University, 103 Borough Road, London SE1 0AA, UK; deym@lsbu.ac.uk (M.D.); dudleyms@lsbu.ac.uk (S.D.); 2 BISITE Research Group, University of Salamanca, Edificio I+D+I, C/ Espejo s/n, 37007 Salamanca, Spain; corchado@usal.es

**Keywords:** indoor localization, received signal strength indicator, fingerprinting, machine learning

## Abstract

Unobtrusive indoor location systems must rely on methods that avoid the deployment of large hardware infrastructures or require information owned by network administrators. Fingerprinting methods can work under these circumstances by comparing the real-time received RSSI values of a smartphone coming from existing Wi-Fi access points with a previous database of stored values with known locations. Under the fingerprinting approach, conventional methods suffer from large indoor scenarios since the number of fingerprints grows with the localization area. To that aim, fingerprinting-based localization systems require fast machine learning algorithms that reduce the computational complexity when comparing real-time and stored values. In this paper, popular machine learning (ML) algorithms have been implemented for the classification of real time RSSI values to predict the user location and propose an intelligent indoor positioning system (I-IPS). The proposed I-IPS has been integrated with multi-agent framework for betterment of context-aware service (CAS). The obtained results have been analyzed and validated through established statistical measurements and superior performance achieved.

## 1. Introduction

Context-aware service (CAS) is gaining attraction due to the proliferation of cellular device use in indoor environments [1,2]. The CAS can be any information, such as indoor location, proximity of devices, place, environmental factors (weather, temperature, time, etc.), status information of devices, behavior of the user (talking, sleeping, walking, etc.), personal fitness, health, etc., to signify the state of the person or user. This information can be extracted from the communication between cellular or mobile device and wireless sensor networks (WSNs) which automatically adapt the environment of a user through an autonomous intelligent agent (AIA). The services are impossible to maintain through satellite based global positioning system (GPS) data when people are inside multistory building, however it is resilient for outdoor areas [3,4]. Crowdsourcing is a positive alternative solution to build ubiquitous indoor positioning systems (IPS) to indoor localization system (ILS) for locate users inside a building [5].

There are several IPS technologies available, such as radio frequency identification (RFID), Ultra Wideband (UWB), infrared (IR), ultrasonic, ZigBee, cellular based, and Bluetooth [6]. The RFID measures proximity and does not need line of sight (LOS) between the RF transmitter and receiver. However, it suffers from a small coverage area, fails to communicate continuously, and cannot be integrated easily with existing infrastructures [7]. UWB systems determine time of arrival (ToA) and time difference of arrival (TDOA) to provide user locations with high accuracy. It can easily handle multipath environments and does not interfere with existing radio frequency (RF) systems, however the presence of metallic materials can cause interference for UWB systems [8]. IR communications use differential phase-shift, angle of arrival (AoA) for positioning but it needs LOS for communication, limiting the capacity within typically small rooms [6]. The ultrasonic based communication does not require LOS but it is highly effected by other high frequency sounds present in that environment [6]. The ZigBee and Bluetooth based communications also do not offer good accuracy for IPS. Cellular devices or Wi-Fi based IPS methods do not interfere with pre-existing frequencies and can be easily integrate with existing infrastructure but, the communication may be effected by signal propagation conditions. Hence, the Wi-Fi based IPS has been chosen for current research which acquired popularity for its ubiquity of 2.4 GHz radio signals where received signal strength indicator (RSSI) information have been considered as probable solution. Localization using received signal strength (RSS) record is executed by two phases: offline and online phase [9,10]. In the offline phase, different features from the transmitted signals in the wireless network are stored at several positions to form a database of location fingerprints. In the online phase, the position is estimated by comparison of the new received values with the database (i.e., with their fingerprint). There are two types of RSSI dependent IPS methods: trilateration and fingerprinting [11]. Wi-Fi trilateration’s goal is to map RSSI as a function of distance. This method requires a steep linear characterization curve in order to be properly implemented. Functions describing these curves are then used with live RSSI values as input to generate an (x,y) location prediction. Wi-Fi Fingerprinting creates a radio map of a given area based on the RSSI data received from several access points and generates a probability distribution of RSSI values for a given (x,y) location. Live RSSI values are then compared to the fingerprint to find the closest match and generate a predicted (x,y) location.

### Contribution

These IPS systems are considered as an AIA among the crowdsourcing services, whereas IPS itself an important service which helps other utilities to achieve broader goals. Therefore, the integration of IPS technology with a multi-agent system (MAS) would be an interesting environment to provide solutions for IPS as well as other positioning depended technologies. Therefore the proposed work aims to form a MAS based intelligent IPS technique for multi-storey buildings. The common ML based IPS approaches have been implemented to obtain possible solution for IPS and *k*-nearest neighbour algorithm has been improved using Jaccard distance measurement where proposed *k*-NN outperforms than existing approaches implemented and discussed in this paper. The contributions of this paper are the following:

1. Several ML techniques have been implemented that allow seamless localization of a smartphone in harsh environments without modifying the existing wireless infrastructure.
2. The use of Jaccard distance in combination with the Nearest Neighbour algorithm has been proposed and outperforms the results obtained with common ML algorithms.
3. The suitability of the proposed approach has been demonstrated by means of a thorough analysis against state-of-the-art ML algorithms applied to the problem addressed.

The rest of the paper is outlined as follows. Fingerprinting localization based works are discussed in Section 2. The current multi-agent architecture proposed by the authors for previous work is described in Section 3, and provides the theoretical description about the proposed framework. The data collection process for this experiment is explained in Section 4, and the evaluation of machine learning algorithms including outcomes are explained in Section 5. Finally, the conclusion is drawn in Section 6.

## 2. Associated Works on IPS

**Figure 1:**
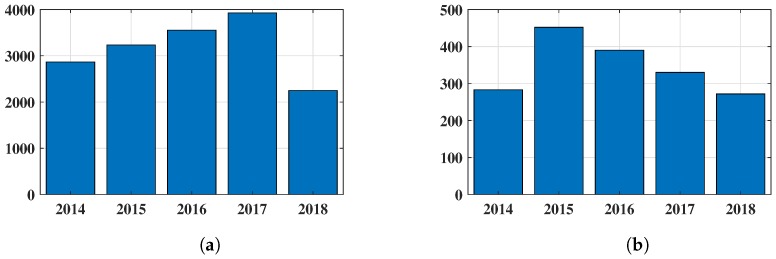
Analysis of publication statistics in the IPS research field: (**a**) publication statistics of IPSs during last five year; and (**b**) publication statistics of IPSs where RSSI has been considered as potential solution.

There have been 15,829 research works published, including article and conference papers between the years 2014 and 2018 (to date) according the Scopus database on indoor localization. The year-wise publication number is shown in Figure 1a for IPS and Figure 1b displays the number of IPS methods where RSSI fingerprinting has been chosen as a cornerstone. The demand for intelligent systems also increase the ML application in this field. Few works have been published where the aspects of IPS solved by ML techniques. Hence, the recent research works of RSSI fingerprinting IPS with ML have been discussed here.

Wu et al. proposed an IPS algorithm using online independent support vector machine (OISVM) learning and undersampling technique to handle the imbalanced data problem. The method employed Wi-Fi RSSI evidence to determine locations. In addition, a kernel function has been implemented for offline training phase for parameter selection [12]. Wang et al. extracted channel state information (CSI) or RSS records to form the IPS tool, PhaseFi. Linear transformation has been applied to discover the bounded variance of each location. This information has been employed to a restricted Boltzmann machine (RBM) to determine weights which have been used along with a greedy learning algorithm in the training phase. Subsequently, a radial basis function (RBF) has been executed for location prediction in the online phase [13]. Fangmin et al. proposed a human tracking algorithm to support elderly people in their daily life. Here, CSI information have been extracted from wireless local area network (WLAN) device. Principle component analysis (PCA) has been implemented to derive the significant information from CSI data, which have been further classified using Forest Decision (FD) method to determine accurate location of elderly people [14]. A fusion based IPS approach was implemented by Liu et al. to handle the complex topology of building and RF transmission where camera, Wi-Fi, and inertial sensors have been unified. It has trained a deep learning method by RSS values of user trajectories in offline phase. The trained algorithm has predicted the scenes in the online stage where the target person is situated and their final position found by using a particle filtering on that scene [15]. In [16], a location based service (LBS) is provided using a decision tree algorithm to offer include directory service, gateway service, location, utility service, presentation service, route service, etc. The decision tree is also used to identify power efficient access points in [17]. The *k*-nearest neighbour algorithm is employed to identify user location inside a building by providing fingerprinting based location profiling techniques in [18,19,20,21,22]. The support vector machine (SVM) is used with different kernels to form efficient indoor navigation system (INS) from Wi-Fi fingerprint and its interaction with existing WSN [23,24,25]. Guo et al. proposed an IPS based on the RSS of visible light LEDs placed on grid points where the received signals power spectral density peaks have been determined; further a least square singular value decomposition (LS-SVD) method has been implemented to mitigate the numerical stability problem and represent the user location through a singular matrix [26]. Baccar et al. created a target map of an environment through fuzzy location indicator (FLI), and collected RSS records according to that FLI. Finally, they fed the RSS values into a neuro fuzzy classifier for indoor location identification [27]. A cost effective IPS was proposed by Yoo et al., who confirm the location including floor without the radio map and positions of Wi-Fi APs. They derived the significant information by feature extraction. The feature representations have been categorized by a Gaussian Process (GP) regression algorithm [28]. Lee et al. created an IPS where the basic service set identity (BSSID) has also been learned along with the RSS record to classify indoor locations. The classification was performed using an ensemble random forest (ERF) method [29]. A similar type RSS classification has been performed in [30] where several popular classifiers are evaluated and the best five classifiers are taken and integrated to implement an IPS application. Xiao et al. proposed a deep learning based scene classification for position identification. The scenes have been gathered from smart phone, and trained the algorithm for online location prediction of users [31].

## 3. Existing Multi-Agent Architecture

**Figure 2:**
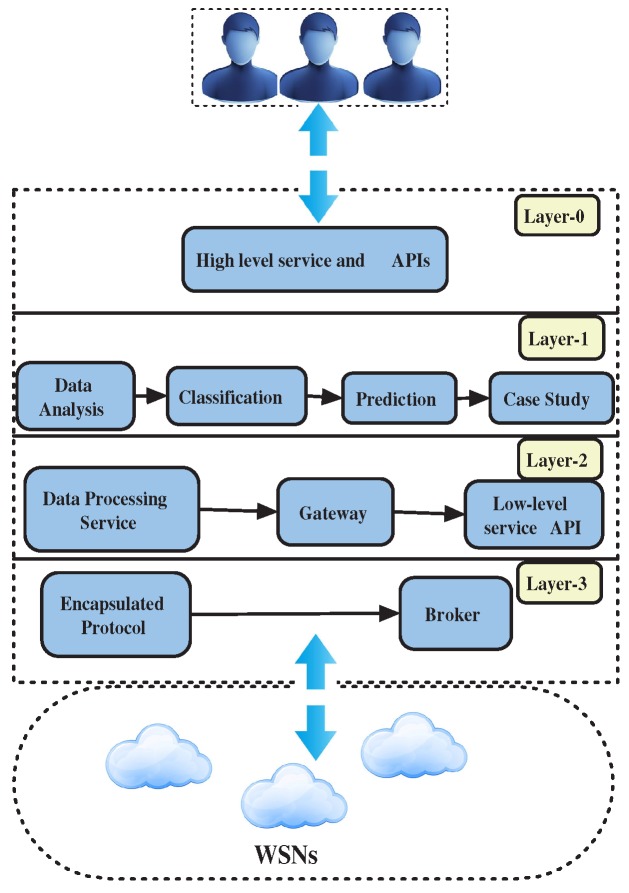
Proposed multi-agent architecture.

The authors built and executed the presented MAS for data fusion and indoor localization, which can be found in [32,33]. This MAS platform has been created with the PANGEA which provides the facility to develop agents and integrate devices [34]. The MAS architecture (shown in Figure 2) has four layers: (a) Layer 0 defines communication with sensor networks of different nature and obtains the raw (encapsulated) data from them; (b) Layer 1 processes the contextual information obtained from Layer 0 and provides a set of low-level services for this purpose; (c) Layer 2 interacts with the agents of Layer 1 and brings other specialized information; and (d) Layer 3 allows the management and customization of services to the end users and facilitates decision-making by the user. The previous work [33] focused on Layer 1 and Layer 2 to process the RSS data to determine location; predictive analysis has been performed in Layer 2 to locate user, and feasibility of ML algorithms for existing architecture. The current research outperforms the previous work by using the Jaccard distance in combination with the Nearest Neighbor algorithm and carries out a thorough analysis against other improved ML techniques. The state-of-the-art has been thoroughly studied to underpin the proposed work where ML algorithms have been implemented for optimal indoor localization outcomes. The offline and online phase have been investigated rigorously to justify the results. In addition, the results have been validated through statistical metrics. The ML decision boundary and the predicted path have been demonstrated in a realistic manner for better understanding. The limitations and future direction are also presented.

## 4. Experimental Set-Up

**Figure 3:**
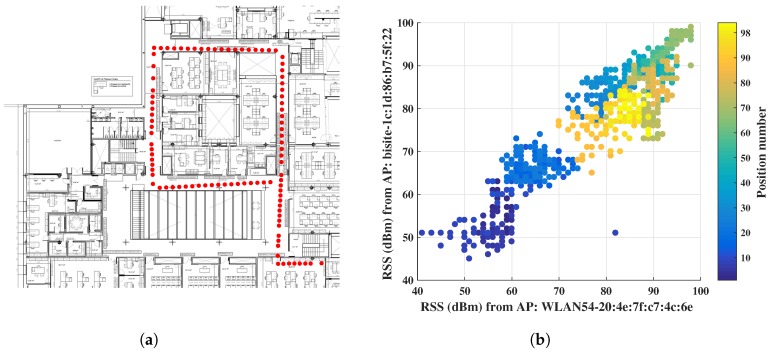
Actual online path obtained from RSS values and their distribution in 2D plane: (**a**) the actual online trajectory; and (**b**) the dots with the same colors are received RSS values at the same position.

The experiment has been conducted through anchors of Cisco Aironet 1600 Series access points (APs) (802.11a/g/n) and a client device LG Nexus 4 (802.11b/g/n) smart phone with Android 5.1.1 Lolipop to collect RSS for our localization experiment. A person was asked to walk along the corridor around different offices and pass through several doors (see Figure 3a) while carrying the mobile to collect RSS from different APs. The corridor path is approximately 120 m long which took approximately 2 min to walk. Most of the APs were located inside the offices while two of them could be seen in the ceiling along the corridor. Therefore, the received signals were highly affected by multipath and non-line-of-sight (NLOS) conditions.

The RSS values were stored in a database along with the known coordinates of the smart phone. A fingerprinting localization database was created by recording a minimum 10 RSS values from all of the detected APs. For example, at location (0,0) the smart phone scanned the Wi-Fi network and stored at least 10 RSS values from every AP detected at that position. In total, 1525 locations with their fingerprints are stored in the database. The proposed ML prototype was trained by these RSS fingerprints to predict the location of any human walking through that path for future tracking or context awareness. The experiment was simulated using Matlab R2017a tool on a Intel${}^{R}$ Core${}^{TM}$ i7 processor@ 3.60 GHz based Windows 7 Enterprise 64 bit operating system with 7856 MB NVIDIA Graphics Processing Unit (GPU). The training dataset was created to develop the model and predict the locations of people in future from RSS fingerprints in offline phase. The model wastested from new RSS values coming from smart phone carried by a different person in the online phase. The online and offline phases are explained in the following sections. Note that the extension of the proposed techniques to the multi-floor case is straightforward since two-dimensional locations can be easily incremented with a third variable referred to the floor (both in the online- and offline phases). The floor in which the user is located will highly affect the RSS values and, therefore, the proposed techniques will easily detect that floor.

## 5. Existing IPS Mechanisms and Outcomes

The proposed RSS fingerprinting model has been considered as multi-class categorization problem for machine learning applications where the coordinates associated with RSS values have been contemplated as class labels to create the ground-truth information. In total, 104 different coordinates were collected with corresponding RSS signatures during the data collection process. These data were used to create the RSS database and train ML algorithms in an offline phase for indoor location prediction. In the online phase, 98 locations along with their corresponding fingerprints were captured among the locations stored in offline database. Initially, the distribution of fingerprinting data was checked to decide upon a suitable ML algorithm. The popular and established ML algorithms of IPS field, such as decision tree [16,17], *k*-NN [18,19,20,21,22], and SVM [23,24,25], were employed to discover the optimal localization performance, but the algorithms achieved very low accuracy. In [18,19], the *k*-NN is investigated with one neighbour (1NN) using Euclidean distance measure, whereas 1NN has been implemented using Spearman Distance or correlation measure in the works of Xie et al. [21] and Yu et al. [22]. The SVMs were employed using two different kernels by following existing works, such as SVM using least squared kernel [23,24] and SVM using Gaussian or radial basis function (RBF) [25], for classifying RSS values. The performance is insignificant to create an intelligent IPS system. As *k*-NN is the most popular and successful ML algorithm in this field, the experiment was continued with *k*-NN to improve the performance and the optimal performance has been achieved through *k*-NN by using Jaccard distance. Therefore, the obtained results from existing algorithms are discussed first and then the performance of improved 1NN using Jaccard distance is detailed. A person was asked to walk in online phase and check the performance of the trained models. The fingerprints of that movement in two-dimensional plane are displayed in Figure 3a by red colored asterisks. In Figure 3b, the RSS values are plotted in a two-dimensional plane to show the distribution of RSS values where *x* and *y* axes express RSS values received from two different APs with their machine address code (MAC). This distribution was used afterwards to analyze prediction results in feature space.

The number of location patterns or coordinates during the walk is *p*, *m* is the number of different coordinates considered as class ${\left\{C_{i}\right\}}_{i=1}^{m}$, and corresponding RSS values ${\left\{y_{j}\right\}}_{j=1}^{p}=\left\{R_{AP_{1}},\;R_{AP_{2}},\;R_{AP_{3}},...,\;R_{AP_{n}}\right\}$ received from *n* different APs for each location are considered as features. The performance of ML algorithms in the online phase are discussed in Table 1 and following sections through standard statistical metrics. The obtained result from different methods were analysed by the achieved accuracy, true positive rate (TPR) or sensitivity, true negative rate (TNR) or specificity, positive predictive value (PPV), and negative predictive value (NPV) [35].

### 5.1. Decision Tree

Decision tree (DT) classifies the RSS values obtained from different locations by forming a tree structure in [16,17]. The model divides the offline RSS training data into smaller subsets based on the ground truths and developed the decision tree for indoor localization. DT represents a top-down approach with decision nodes and leaf nodes within leaf nodes added as coordinates decided by the RSS decision rules. It begins with available RSS values to divide upon the classes. First, the most significant source of RSS values, i.e., AP from offline data, is decided by calculating entropy and information gain. The entropy is determined as $H\left(R_{AP_{1}}\right)=-\sum_{}^{}p\left(R_{AP_{1}}\right)\log p\left(R_{AP_{1}}\right)$ where $p(R_{AP_{1}})$ is probability of a RSS value $R_{AP_{1}}$ which comes from access point $AP_{1}$, and information gain $IG=H\left(R_{AP_{1}}\right)-H\left(T,\;R_{AP_{1}}\right)$ where *T* is a target coordinate. All received RSS values are sorted with their corresponding AP. Then, the AP with the highest information gain is placed at the root position. Now, this process is recursively implemented to reach target coordinate through the RSS values and make the full decision tree. After successful implementation of decision rules in offline or training phases, new sequences of RSS values are employed to the system for indoor location prediction.

**Figure 4:**
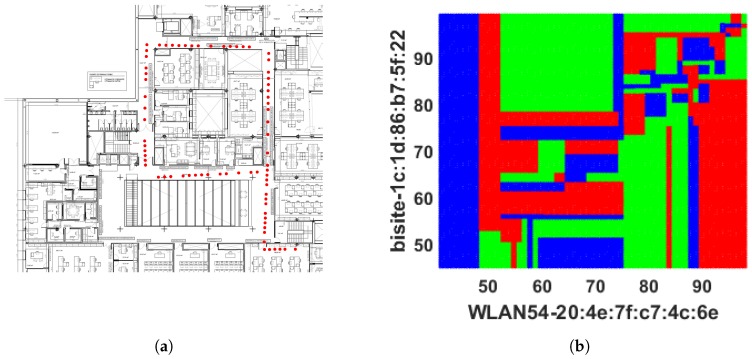
Prediction results obtained from DT classifier: (**a**) the route predicted by DT classifier; and (**b**) 2D decision boundary formed by DT classifier.

**Figure 5:**
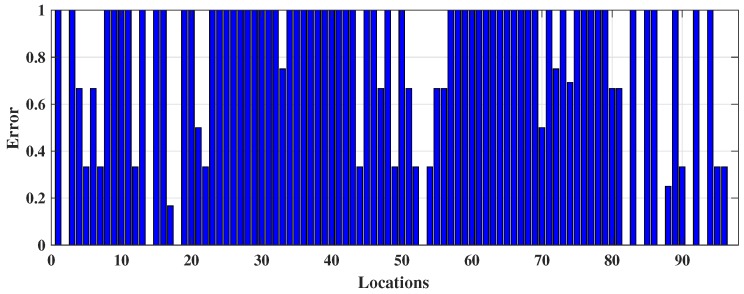
The occurrence of error in each coordinate from DT classification during online phase.

The prediction outcomes are listed in Table 1. It has predicted 45.36% (≡0.4536) locations correctly, which demonstrates the model can predict approximately 45 positions correctly including true positive (TP) and true negative (TN) out 100. Here, TP indicates the number locations that have been truly predicted by the model and TN is the number of locations that do not belong to a target location have been correctly identified. The sensitivity or TPR 54.64% (≡0.5464) indicates approximately 55 locations out 100 have been detected correctly. Specificity or TNR refers the capability of identifying approximately 62 (≡0.6186) locations out of 100 which do not belong to a class or those locations belong to a different class. The low PPV and high NPV refer probability of prediction that locations get negative result truly do not belong to a class. The outcomes show DT has made a number of misidentifications because it forms a tree structure based on the offline RSS data; however, when new data arrive during the online phase, they fail to fit under the defined DT rules, causing sampling errors, leading to the DT model delivering weak performance. The locations identified by the DT model is shown in Figure 4a to demonstrate where the misclassification happened by DT whereas, the actual online locations (shown in Figure 3a) are more continuous. In addition, Figure 4b expresses the two-dimensional decision boundary created by DT, whereas Figure 3b indicates the distribution of that RSS data in a two-dimensional plane. Figure 5 shows the errors occurred in each location during the online testing phase where the *x* axis denotes the locations (or the coordinates) acquired and *y* axis symbolizes the error determined for the respective coordinate location. It hwas found that maximum errors appeared between Class 20 and Class 80.

### 5.2. *k*-Nearest Neighbour Models

The nearest neighbour model is simple and effective ML algorithm in fingerprinting classification domain. Thus, different *k*-NN models [18,19,20,21,22] were implemented and their outcomes analyzed.

#### 5.2.1. $1NN_{Eu}$

The conventional *k*-NN (using Euclidean distance) is employed with one nearest neighbour $1NN_{Eu}$ in [18,19] to recognize the RSS patterns obtained from different locations. If a new set of RSS value in online phase is $x=\{R_{AP_{1}}^{new},\;R_{AP_{2}}^{new},\;R_{AP_{3}}^{new},...,\;R_{AP_{n}}^{new}\}$ for a location, then 1NN algorithm measures distance between *x* and a priori knowledge database ${\left\{y_{j}\right\}}_{j=1}^{p}$ using $||x-y_{j}\left|\right|=\max_{1\leq j\leq p}||x-y_{j}\left|\right|$ to decide the class or location coordinate of $x\in C_{i}$ where $C_{i}$ represents the class or location of RSS patterns stored in database. The outcomes are listed in Table 1. This model achieved only 46.77% (≡0.4677) accuracy with high TPR, however TNR is very low in this case.

#### 5.2.2. $1NN_{Sp}$

$1NN_{Eu}$ was modified using Spearman distance, i.e., $1NN_{Sp}$, to obtain improved performance. This model is used in [21,22] to handle the multipath problem and classify locations through RSS values. Here, 1NN are used and distance between RSS patterns are measured by Spearman correlation. The correlation between *x* and $y_{j}$ are determined by $Co=\frac{cov(r_{x},{r_{y}}_{j})}{{\sigma_{r}}_{x},{{\sigma_{r}}_{y}}_{j}}$ where $r_{x}$ and ${r_{y}}_{j}$ are the ranks of *x* and $y_{j}$, respectively, cov is covariance of the ranks, and ${\sigma_{r}}_{x}$ and ${{\sigma_{r}}_{y}}_{j}$ are standard deviations of the ranks $r_{x}$ and ${r_{y}}_{j}$, respectively. The best correlation of +1 or −1 is obtained when each stored RSS pattern is perfect monotone function of the incoming RSS patterns. The results obtained from this model are shown in Table 1. The outcomes are similar as $1NN_{Eu}$. The accuracy 48.56% (≡0.4856) is insignificant for an automated IPS framework. Here, the correlation uses the ranking of the RSS values where the significant variations of fingerprints cannot be measured as long as the order remains same and correlation coefficient will be same. This drawback prevents $1NN_{Sp}$ to be considered as an efficient model for IPS in this case.

#### 5.2.3. $3NN_{Eu}$

**Figure 6:**
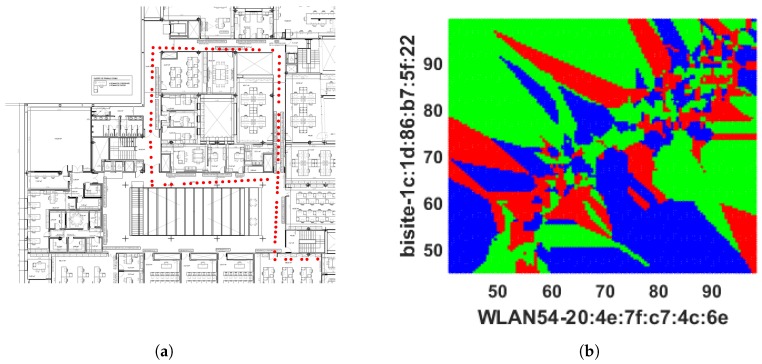
Prediction results obtained from 3NN${}_{Eu}$ classifier: (**a**) the route predicted by 3NN${}_{Eu}$ classifier; and (**b**) 2D decision boundary formed by 3NN${}_{Eu}$ classifier.

**Figure 7:**
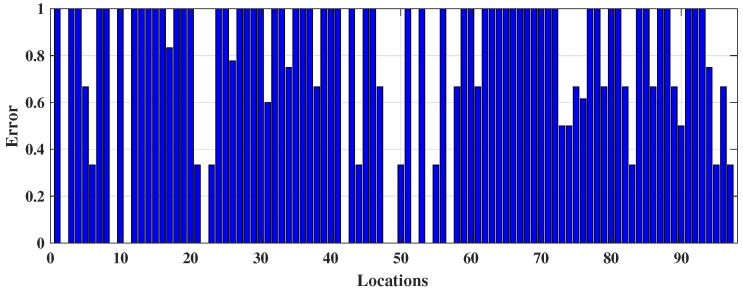
The occurrence of error in each coordinate from 3*NN*${}_{Eu}$ classification during online phase.

The number of nearest neighbour *k* has been modified to enhance the majority rule which was executed by Cheong et al. [20] to better identify the indoor locations from Wi-Fi fingerprinting and integrate that information with a GPS system via FPGA embedded technology. It employs three (k=3) nearest neighbour and distance between stored and online fingerprints are determined by Euclidean distance, i.e., $3NN_{Eu}$. Table 1 shows that it performed slightly better than DT, $1NN_{Eu}$, and $1NN_{Sp}$ but had a high number of false predictions. It achieved only 48.78% (≡0.4878) accuracy. The TPR or sensitivity of 11.06% (≡0.1106) specifies that it can only truly predict approximately 11 locations out of 100, and TNR or specificity 68.73% (≡0.6873) indicates that it can predict approximately 69 responses correctly out of 100 to illustrate the negative prediction which means 69 times the 3NN${}_{Eu}$ model is right when it predicts an incoming RSS pattern does not specify a location. In addition, 3NN${}_{Eu}$ induces very low PPV which indicates a high probability of location misidentification. The reason for misclassifications in this case arises from the negative sign of the fingerprints (in dBm unit) which is squared by the model during distance measurement makes a low RSS value dominant at the time of classification and reduces the significance of a RSS pattern. Additionally, fingerprints are affected by multipath noise interference which creates difficulties for obtaining better localization outcomes. However, 3NN${}_{Eu}$ performed better than the other *k*-NN models implemented here. Thus, the movement path of the participants online phase predicted by 3NN${}_{Eu}$ is included in Figure 6a. It demonstrates that, bottom left, left, and top-right positions are misclassified mostly and the locations (or coordinates) overlapped by other co-ordinates which results a visually discrete path. Figure 6b displays the decision boundary predicted by 3NN${}_{Eu}$ to show the difference between expected boundary (from Figure 3b) and the predicted decision boundary. Figure 7 shows the error obtained in each location during online phase where *x* axis denotes the locations (or the coordinates) gathered and *y* axis signifies the error calculated for respective coordinate location.

### 5.3. Support Vector Machine Models

The SVM is one of most powerful and well known ML technique famous for its kernel functions. The optimized hyperplane could be achieved by solving different functions. There are two SVM models have been tested from [23,24,25] and presented in the following sections.

#### 5.3.1. $SVM_{LS}$

The Least Square-Support Vector Machines (LS-SVM) is investigated in [23,24] to obtain human location from different types of motion (e.g., static, standing with hand swinging, normal walking while holding the phone in hand, etc.) using RSS fingerprints. In Figure 3b, the data are non-linearly separable, which motivates the use of LS-SVM or $SVM_{LS}$. It solves set of linear equations to find an optimized margin for fingerprints in the hyperspace. The $SVM_{LS}$ forms the margin minimization problem as $min\left\{J\left(w,b,e\right)\right\}=\frac{\mu }{2}w^{T}w+\frac{\zeta }{2}\sum_{i=1}^{N}{e_{i}}^{2}$ with an equality constraints $y_{i}\left[w^{t}\phi \left(x_{i}\right)+b\right]=1-e_{i}$ where *J* is the cost function, *w* is weight vector, *b* is the bias, *e* is the positive slack variable, μ and ζ are hyper-parameters to tune the regularization process, and $\phi (x_{i})$ and $y_{i}$ are the RSS fingerprints and their class labels (coordinates), respectively. It delivers better accuracy and other metric value than decision tree and NN based models (in Table 1). It achieved accuracy of 60.70% (≡0.6070). In addition, $SVM_{LS}$ produces good TNR of 68.04% (≡0.6804) and high NPV of 98.51% (≡0.9851) in this case. However, it results low TPR and PPV values, showing weak performance in identifying the true class of fingerprints.

#### 5.3.2. $SVM_{RBF}$

Subsequently, the SVM was investigated with Gaussian kernel or radial basis function to obtain better indoor localization result and SVM with Gaussian kernel or radial basis function, i.e., SVM${}_{RBF}$. This model is used in [25] to locate a user along with device for betterment of ubiquitous computing services (UCS). The discriminant function of SVM${}_{RBF}$ is designed as, $f\left(x\right)=\sum_{j=1}^{p}\alpha_{j}k\left(y_{j},y\right)+b$ and $k\left(y_{j},y\right)=exp(-||y-y_{j}{\left|\right|}^{2}/2\sigma^{2})$ where $\alpha ,\;y_{j},\;y,\;\sigma ,\;b$ are weight, support vector, offline RSS values (training vector), free parameter, and bias, respectively. The term $||y-y_{j}{\left|\right|}^{2}$ is realized as squared Euclidean distance between an offline RSS fingerprint and the support RSS fingerprint to decide the hyperplane for classification. The $1/2\sigma^{2}$ is a priori knowledge and considered as greater than zero. Once the margin of separation is maximized, the weight vectors are used for prediction based on offline RSS data. As the RSS values are received from different APs for each location, some RSS values are missing at particular positions because of the weak signal strength creating a heterogeneous RSS set for some coordinates. Therefore, the missing values are replaced with zero. Now, the SVM${}_{RBF}$ uses the dot product between features (RSS values), thus the products become zero in that case, and adjustment of weight become impossible for such coefficients. Hence, the respective classes (coordinates) are misclassified by the SVM${}_{RBF}$.

**Figure 8:**
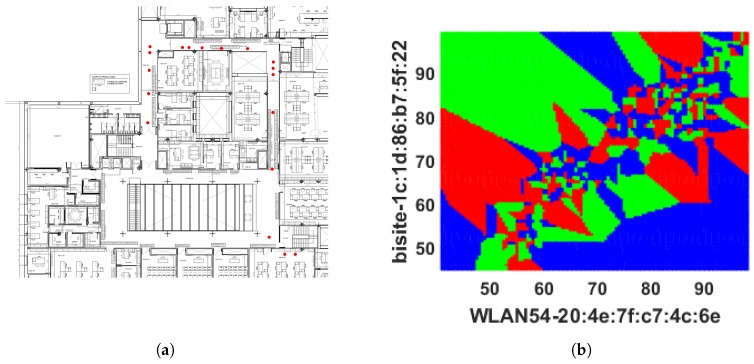
Predicted Paths by SVM: (**a**) the route predicted by SVM${}_{RBF}$ classifier; and (**b**) 2D decision boundary formed by SVM${}_{RBF}$ classifier.

**Figure 9:**
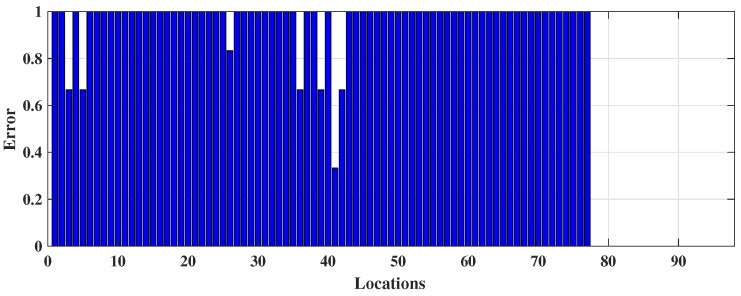
The occurrence of error in each coordinate from SVM${}_{RBF}$ classification during online phase.

The outcomes of SVM${}_{RBF}$ are listed in Table 1 and it was found that it failed to predict locations sufficiently. Although it delivered an accuracy of 64.29% (≡0.6429), the TPR of 19.01% (≡0.1901) indicates the model predicts only 19 locations correctly out of 100 and a TNR of 4.81% (≡0.0481) specifies that the model can only correctly predict 5 negative predictions out of 100. This misidentification leads to very low positive prediction probability. This model performed better among the two SVMs, thus the outcomes of SVM${}_{RBF}$ are included here. Figure 8a shows that very few locations have been determined by SVM${}_{RBF}$ where some locations have overlapped by others because of the misclassifications and visually it looks like a discrete path. In addition, the decision boundary predicted by SVM${}_{RBF}$ is included in Figure 8b. Figure 9 shows the error determined in each location during the online phase where the *x* axis denotes the locations (or the coordinates) gathered and the *y* axis signifies the error calculated for respective coordinate location. The misclassification of locations by SVM${}_{RBF}$, as discussed above, is displayed in more detail here.

### 5.4. Improved *k*-NN and Outcomes

The conventional *k*-NN model has been improved using one nearest neighbour and Jaccard distance, i.e., 1NN${}_{Ja}$. The Jaccard similarity coefficient is explained as the size of the intersection divided by the size of union of the stored and online fingerprint set $J\left(x,y_{j}\right)=\frac{|x\cap y_{j}|}{|x\cup y_{j}|}$. It is complementary to subtract the Jaccard coefficient from 1 to determine the similarity. The model 1NN${}_{Ja}$ has achieved better outcomes than all other ML algorithms implemented here and results, as shown in Table 1. The Jaccard distance does not have equal priority to positive and negative numbers, unlike Euclidean norms, which helps to discover similarities and dissimilarities among RSS patterns more clearly. In addition, only one number (k=1) of NN fits well for location prediction here.

**Figure 10:**
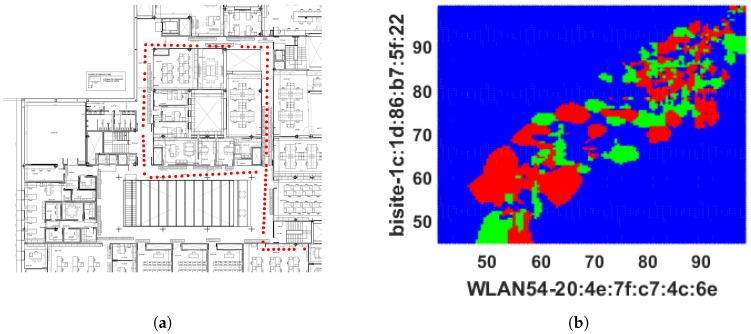
Prediction results obtained from 1NN${}_{Ja}$ classifier: (**a**) the route predicted by 1NN${}_{Ja}$ classifier; and (**b**) 2D decision boundary formed by 1NN${}_{Ja}$ classifier.

**Figure 11:**
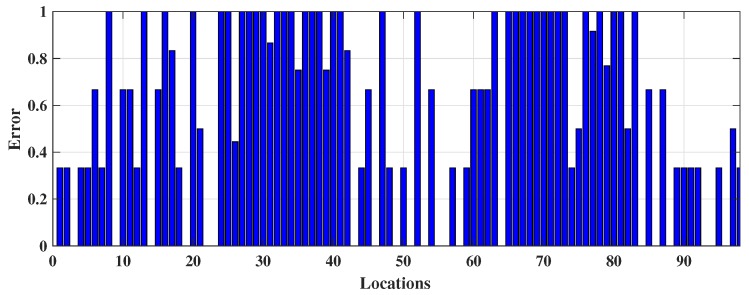
The occurrence of error in each coordinate from 1NN${}_{Ja}$ classification during online phase.

The 1NN${}_{Ja}$ model delivered accuracy of 78.84% (≡0.7884). In addition, the TPR of 96.67% (≡0.9697) indicates that approximately 97 times 1NN${}_{Ja}$ gives positive responses out of 100 which is correct and a TNR of 97.35% (≡0.9735) indicates approximately 97 times the model provides negative decision which is correct. Better TPR and TNR values also increase the probability of positive and negative prediction. Figure 10a expresses the locations predicted by 1NN${}_{Ja}$ are better than the path have been predicted by the other ML algorithms (DT, 3NN${}_{Eu}$, and $SVM_{RBF}$) here. In addition, the decision boundary is included in Figure 10b, which indicates that the RSS patterns have been classified appropriately by 1NN${}_{Ja}$. Figure 11 presents the error occurred in each location during online phase where *x* axis denotes the locations (or the coordinates) gathered and *y* axis signifies the error calculated for respective coordinates. It was found that errors occurred for few locations, which reflects the overall performance displayed in Table 1.

### 5.5. Result Comparison

**Figure 12:**
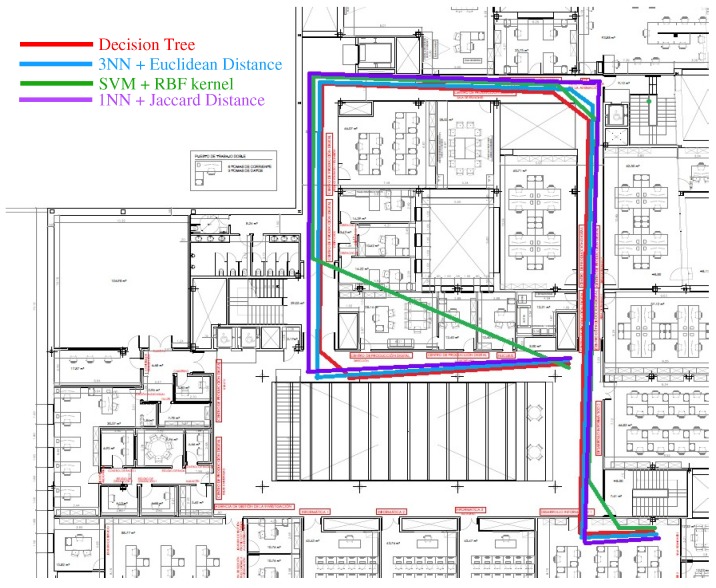
Comparison of predicted paths attained from different ML algorithms.

**Figure 13:**
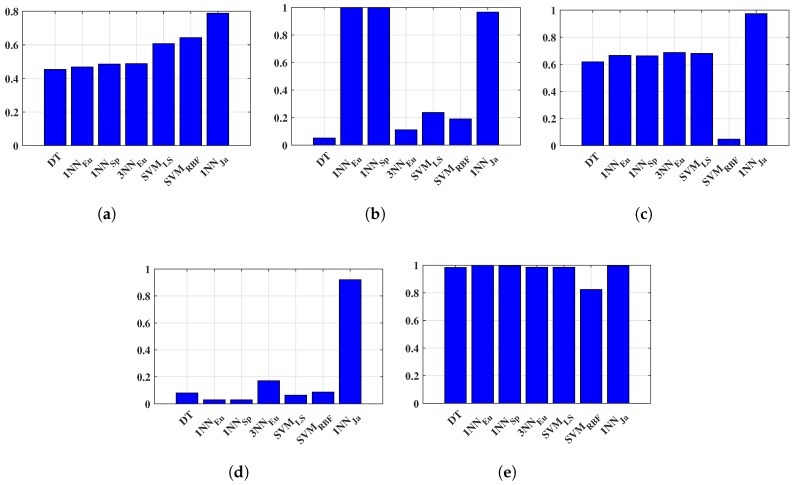
Comparison of proposed methods based on different statistical metrics: (**a**) accuracy; (**b**) true positive rate; (**c**) true negative rate; (**d**) positive predictive value; and (**e**) negative predictive value.

The obtained paths and statistical metrics are compared in this section. The classified locations or coordinates are discussed and shown in Figure 4a, Figure 6a, Figure 8a, and Figure 10a. Figure 12 shows the walking routes acquired by connecting those locations. In addition, it demonstrates the performance of improved *k*-NN ($1NN_{Ja}$) along with existing ML algorithms (DT, $3NN_{Eu}$, $SVM_{RBF}$, and $1NN_{Ja}$) in real life indoor scenario. The existing and proposed frameworks have both been validated through statistical metrics, as discussed above. Those metrics attained for all algorithms are displayed in Figure 13 to compare the performance of the ML algorithms more appropriately. Figure 13a–e presents the comparison of methods based on accuracy, TPR or sensitivity, TNR or specificity, PPV, and NPV, respectively. The proposed 1NN${}_{Ja}$ delivered the optimal performances for making an I-IPS in terms of predicted route and metrics.

## 6. Conclusions and Future Work

A MAS based intelligent IPS has been proposed. The results have been validated and a predicted path has been executed via a building floor for understanding the results as well as the route of a user in the online phase. In addition, the actual path and predicted path both have been plotted during the evaluation of ML classifiers. The authors have tried to achieve accurate locations by implementing ML classifiers where *k*NN produced optimal indoor localization results, in terms of statistical validation metrics. However, RSS records are imbalanced for some coordinates which means the RSS values of APs are missing in a small number of places. It leads to a data or feature imbalance in the classification phase where some coordinates dominate others. To overcome this problem, feature will be extracted from the RSS values instead of using raw RSS values in future. In addition, further ML algorithms will be explored with parameter tuning to create more effective and successful MAS based intelligent IPS.

## Figures and Tables

**Table 1:** Comparison of results obtained from different ML methods.

Classifiers	Accuracy	TPR	TNR	PPV	NPV
Decision Tree [16,17]	0.4536	0.0520	0.6186	0.0805	0.9836
1NN+Euclidean Distance [18,19]	0.4677	0.9988	0.6667	0.0300	0.9995
1NN+Spearman Distance [21,22]	0.4856	0.9978	0.6632	0.0297	0.9945
3NN + Euclidean Distance [20]	0.4878	0.1106	0.6873	0.1714	0.9852
SVM + Least Squared Kernel [23,24]	0.6070	0.2377	0.6804	0.0641	0.9851
SVM + Guassian Kernel [25]	0.6429	0.1901	0.0481	0.0866	0.8235
1NN + Jacard Distance	0.7884	0.9667	0.9735	0.9206	0.9949
